# Magnesium Transporter MGT6 Plays an Essential Role in Maintaining Magnesium Homeostasis and Regulating High Magnesium Tolerance in *Arabidopsis*

**DOI:** 10.3389/fpls.2018.00274

**Published:** 2018-03-12

**Authors:** Yu-Wei Yan, Dan-Dan Mao, Lei Yang, Jin-Liang Qi, Xin-Xin Zhang, Qing-Lin Tang, Yang-Ping Li, Ren-Jie Tang, Sheng Luan

**Affiliations:** ^1^Department of Plant and Microbial Biology, University of California, Berkeley, Berkeley, CA, United States; ^2^Key Laboratory of Biology and Genetic Improvement of Maize in Southwest Region, Maize Research Institute of Sichuan Agricultural University, Chengdu, China; ^3^Nanjing University–Nanjing Forestry University Joint Institute for Plant Molecular Biology, State Key Laboratory for Pharmaceutical Biotechnology, College of Life Sciences, Nanjing University, Nanjing, China; ^4^College of Life Sciences, Hunan Normal University, Changsha, China; ^5^Key Laboratory of Saline-Alkali Vegetation Ecology Restoration in Oil Field, Ministry of Education, Alkali Soil Natural Environmental Science Center, Northeast Forestry University, Harbin, China; ^6^Key Laboratory of Horticulture Science for Southern Mountainous Regions, Southwest University, Chongqing, China

**Keywords:** Mg^2+^ transporter, Mg^2+^ homeostasis, *Arabidopsis*, MGT6, MGT7

## Abstract

Magnesium (Mg) is one of the essential nutrients for all living organisms. Plants acquire Mg from the environment and distribute within their bodies in the ionic form via Mg^2+^-permeable transporters. In *Arabidopsis*, the plasma membrane-localized magnesium transporter MGT6 mediates Mg^2+^ uptake under Mg-limited conditions, and therefore is important for the plant adaptation to low-Mg environment. In this study, we further assessed the physiological function of MGT6 using a knockout T-DNA insertional mutant allele. We found that MGT6 was required for normal plant growth during various developmental stages when the environmental Mg^2+^ was low. Interestingly, in addition to the hypersensitivity to Mg^2+^ limitation, *mgt6* mutants displayed dramatic growth defects when external Mg^2+^ was in excess. Compared with wild-type plants, *mgt6* mutants generally contained less Mg^2+^ under both low and high external Mg^2+^ conditions. Reciprocal grafting experiments further underpinned a role of MGT6 in a shoot-based mechanism for detoxifying excessive Mg^2+^ in the environment. Moreover, we found that *mgt6 mgt7* double mutant showed more severe phenotypes compared with single mutants under both low- and high-Mg^2+^ stress conditions, suggesting that these two MGT-type transporters play an additive role in controlling plant Mg^2+^ homeostasis under a wide range of external Mg^2+^ concentrations.

## Introduction

Magnesium (Mg) is an essential macronutrient for plants. Being the most abundant free divalent cation in living cells, Mg^2+^ serves as a counter ion for nucleotides and a central metal for chlorophylls, and acts as a cofactor for many enzymes in catalytic processes. Mg^2+^ also contributes to membrane stabilization and active conformation of macromolecules (Shaul, 2002). Both low and high levels of Mg present in the soil are deleterious to plant growth, thus affecting crop production. Due to unbalanced application of chemical fertilizers, plants may exhibit Mg deficiency symptoms in the presence of high levels of other cations such as calcium (Ca^2+^) and potassium (K^+^) in the soil (Hermans et al., 2013). Moreover, excessive aluminum (Al^3+^) in acidic soils or other heavy metals severely inhibit the uptake of Mg^2+^, resulting in Mg deficiency in the plants. These problems lead to reduction in crop yield as well as higher susceptibility to some plant diseases. On the other hand, high levels of Mg are found in serpentine soils featuring a low Ca/Mg ratio (Brady et al., 2005). Genome sequencing of *Arabidopsis lyrata* plants grown in serpentine or non-serpentine habitats has identified a number of polymorphisms associated with Ca^2+^ and Mg^2+^ transport (Turner et al., 2010). Although it is critical for plant cells to maintain an optimal Mg^2+^ level for normal growth and development, the transport and regulatory mechanisms that govern Mg^2+^ acquisition, distribution, and reallocation are poorly understood (Tang and Luan, 2017).

In bacterial cells, there are at least three distinct types of membrane proteins CorA, MgtE, and MgtA/B that are capable of transporting Mg^2+^. While the MgtE channel and the P-type ATPases MgtA/B do not seem to have any close homologs in plants, there is a major family of Mg^2+^ transporters (MGTs) related to bacterial CorA proteins (Li et al., 2001). They are also named as “MRS2s” based on the ability to rescue the yeast *mrs2* mutant lacking the Mrs2 protein, a yeast homolog of CorA-type transporter that mediates Mg^2+^ transport into the mitochondrial matrix (Schock et al., 2000). The CorA-family proteins feature a unique topology with two closely spaced, C-terminal transmembrane (TM) domains, the first of which contains a conserved GMN (Gly-Met-Asn) tripeptide motif that is essential for Mg^2+^ transport (Szegedy and Maguire, 1999). Crystal structure of the *Thermotoga maritima* CorA establishes the protein as a pentameric cone-shaped ion channel (Eshaghi et al., 2006; Lunin et al., 2006).

Several members of the *Arabidopsis* MGTs facilitate Mg^2+^ transport in bacteria or yeast (Li et al., 2001, 2008; Mao et al., 2008, 2014; Gebert et al., 2009). Genes coding for MGT-type transporters are widely expressed in various plant tissues and cell types in *Arabidopsis* (Li et al., 2001; Gebert et al., 2009) and the proteins are targeted to plasma membrane or intracellular membranes, implicating MGT members functioning in Mg^2+^ transport across multiple cellular membranes. MGT1 is mainly expressed in the root hair and the elongation zone as well as the vascular tissues and leaf trichomes (Gebert et al., 2009), suggesting a potential role in Mg^2+^ translocation in these particular cell types. MGT2 and MGT3 are associated with vacuolar membrane and possibly involved in Mg^2+^ homeostasis in leaf mesophyll cells (Conn et al., 2011). Quite a few MGTs including MGT4, MGT5, and MGT9 are highly expressed in pollen and anther cells, and are required for plant reproduction, suggesting that active Mg^2+^ transport is critical for pollen development (Li et al., 2008, 2015; Chen et al., 2009; Xu et al., 2015). MGT10 is localized in the chloroplast envelope, and is strongly expressed in the rosette and cauline leaves, indicating its possible function in Mg^2+^ translocation into chloroplasts (Drummond et al., 2006). Indeed, two recent studies confirmed that mutant plants lacking MGT10 show defects in chloroplast development and plant photosynthesis (Liang et al., 2017; Sun et al., 2017). In rice, OsMGT1 is localized to the plasma membrane and its rapid up-regulation upon Al^3+^ stress confers Al^3+^ tolerance on rice plants as a result of enhanced Mg^2+^ uptake (Chen et al., 2012). Interestingly, OsMGT1 plays a role in rice salt tolerance possibly through activating the transport activity of OsHKT1;5 (Chen et al., 2017).

Among all the MGT-type Mg^2+^ transporters in *Arabidopsis*, MGT6 and MGT7 are thought to be more directly involved in controlling cellular Mg^2+^ homeostasis because impairment of MGT6 or MGT7 function renders *Arabidopsis* plants hypersensitive to low-Mg conditions (Gebert et al., 2009; Mao et al., 2014; Oda et al., 2016). MGT6 appears to be localized to the plasma membrane and mediate the high-affinity Mg^2+^ uptake via roots (Mao et al., 2014). Consistent with this role, expression of *MGT6* is dramatically up-regulated at the transcriptional level when external Mg^2+^ becomes limited (Mao et al., 2014). MGT7 is preferentially expressed in roots, and also plays an important role for plant adaptation to low-Mg conditions although the mechanism is not clear (Gebert et al., 2009). In this study, we showed that MGT6 is equally important for controlling plant Mg^2+^ homeostasis under normal and high-Mg conditions. We uncovered a shoot-based mechanism that underlies MGT6 function in detoxifying excessive Mg^2+^, in addition to its role in root Mg^2+^ uptake under Mg-limited conditions. Furthermore, by analyzing the *mgt6 mgt7* double mutant, we showed that these two Mg^2+^ transporters MGT6 and MGT7 play an overlapping role in maintaining essential Mg^2+^ homeostasis under a wide range of external Mg^2+^ concentrations.

## Materials and Methods

### Plant Materials and Growth Conditions

*Arabidopsis thaliana* ecotype Col-0 was used in this study. T-DNA insertional mutant lines were obtained from the *Arabidopsis* Biological Resource Center. The seed stock IDs are as follows: SALK_205483 (*mgt6*) and SALK_064741 (*mgt7*). The double mutant *mgt6 mgt7* was generated by crossing *mgt7* to *mgt6* mutant, and progeny of F2 generation was screened for double homozygous mutations in *MGT6* and *MGT7* using a PCR-based genotyping approach.

Wild-type and mutant plants were grown in the soil at 22°C under the 16-h-light/8-h-dark condition in the greenhouse. Hydroponically grown plants were generally kept in the 1/6 strength MS solution under the short-day condition (8-h-light/16-h-dark) in the greenhouse. Fresh liquid solutions were replaced twice a week.

### Phenotypic Assays

*Arabidopsis* seeds of different genotypes were sterilized with 10% bleach for 5 min and washed in sterilized water for 3 times. Seeds were sown on the solid plates supplemented with different concentrations of Mg^2+^. The basal medium contained 1/6 strength of MS salt (Murashige and Skoog, 1962) in which MgSO_4_ was replaced by the K_2_SO_4_. Different concentrations of MgCl_2_ were added as the Mg^2+^ source. After 2-day stratification at 4°C, plates were vertically grown at 22°C in the growth chamber.

For the post-germination assay, seeds were first sown on MS medium solidified with 1% phytoagar. After germination, 5-day-old seedlings were transferred onto 1/6 Mg^2+^-free MS medium (containing 1% sucrose, pH = 5.8, solidified with 0.8% agarose) supplemented with Mg^2+^ at the indicated concentrations.

For phenotypic assay in the hydroponics, 7-day-old seedlings were transferred to liquid solutions containing 1/6 MS salts supplemented with 1.25 mM MgSO_4_. After 2-week culture, the plants were treated with solutions containing different concentrations of Mg^2+^.

### Functional Complementation

For complementation of the *mgt6* mutant, a 3.5-kb genomic fragment including the *MGT6* coding region as well as 1.5 kb of the 5′ flanking DNA upstream of the starting codon was amplified by PCR from *Arabidopsis* genomic DNA with forward (5′-ACGGATAAATGTGGGGATGCTTG-3′) and reverse (5′-CCAAATCAAATCAACCCATAAAC-3′) primers. The PCR product was cloned into the SmaI site of the binary vector pCAMBIA1300. After sequencing, the construct was transformed into *Agrobacterium tumefaciens* strain GV3101 and introduced into *mgt6* mutant plants by the floral dip method (Clough and Bent, 1998). Transgenic seeds were screened on MS medium supplemented with 25 mg/L hygromycin. Resistant seedlings were transplanted to soil and grown in the greenhouse for seed propagation. T3 homozygous transgenic plants were subject to gene expression analysis and phenotypic assays together with wild-type plants and *mgt6* mutants.

### RNA Isolation and Gene Expression Analysis

Total RNA was extracted from plant materials using the TRIzol reagent (Invitrogen). After being digested by DNase I (Invitrogen) to decontaminate DNA, cDNA was generated from RNA samples at 42°C using SuperScript II reverse transcriptase (Invitrogen). The resultant cDNA samples were used for PCR amplification with the gene-specific primers. Quantitative real-time PCR was performed on the DNA Engine Opticon System (MJ Research) using the SYBR Green Realtime PCR Master Mix to monitor double-stranded DNA products. Data were calculated based on the comparative threshold cycle method. The relative expression of each Mg-starvation marker gene was double-normalized using the housekeeping gene *ACTIN2* and using the control expression values measured in the wild type when external Mg^2+^ is 1.5 mM.

### Grafting Experiments

Reciprocal grafting experiments were performed as previously described with minor modifications (Marsch-Martínez et al., 2013). Seeds were sown on MS medium containing 1% agar and 2% sucrose, and grown vertically in the growth chamber (22°C, 14-h-light/10-h-dark) after 2-day stratification at 4°C. Six-day-old *Arabidopsis* seedlings were transversely cut with a sharp blade in the middle position of the hypocotyl so that each individual seedling was divided into two parts. Subsequently, different parts of each material were re-assembled and grafted on half MS medium supplemented with 1.2% agar, 0.5% sucrose, 3 mg/L Benomyl [methyl 1-(butylcarbamoyl)-2-benzimidazolecarbamate], 0.02 mg/L IAA (indole acetic acid) and 0.04 mg/L 6-BA (6-benzylaminopurine). The grafted seedlings were grown vertically in the growth chamber for another 10 days to allow the formation of the graft union. Successfully unified seedlings with the same size and status were then transferred to the hydroponic culture for further experiments.

### Measurement of the Mg and Ca Content

Plant samples were harvested from root and shoot tissues, respectively, and briefly washed with ddH_2_O for 10 s. The samples were then thoroughly dried up in the oven at 80°C. The dry matters were collected in the 15 mL centrifuge tubes (ions free) and digested with 1 mL ultrapure HNO_3_ (Sigma-Aldrich) in the water bath at 95°C for 4 h. Digested samples were diluted to the appropriate concentrations with ddH_2_O, and the elemental concentrations were determined by inductively coupled plasma optical emission spectroscopy (ICP-OES; PerkinElmer, Waltham, MA, United States).

## Results

### Knockout Mutation in MGT6 Leads to Plant Hypersensitivity to Mg Deficiency

**FIGURE 1:**
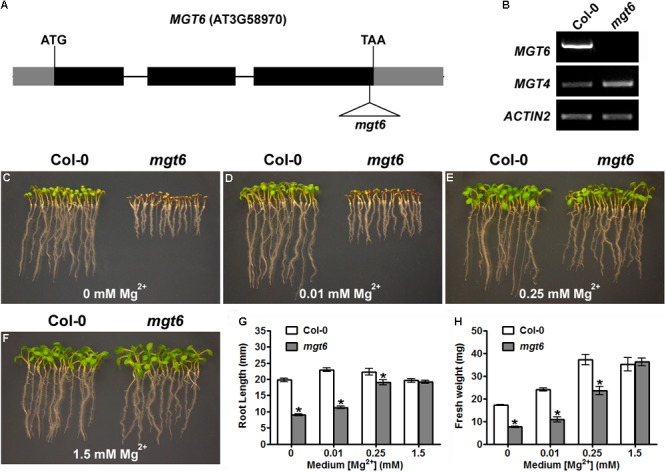
The T-DNA insertional *mgt6* mutant is hypersensitive to Mg^2+^ deficiency. **(A)** Schematic diagram of the T-DNA insertion in the *mgt6* mutant. Exons and introns are depicted to scale by boxes and lines, respectively. The coding region of the gene is shown as black boxes while the 5′ and 3′ UTR of the cDNA is shown as light-shaded boxes. The position of the T-DNA is indicated by the triangle. **(B)** RT-PCR analysis of *MGT6* and *MGT4* in wild-type and *mgt6* seedlings. **(C–F)** Growth phenotype of young seedlings 6 days after seed germination on the medium supplemented with different concentrations of Mg^2+^. **(G)** Quantification of root length of 7-day-old seedlings. **(H)** Quantification of seedling fresh weight. Data represent means ± SE of four replicate experiments. Asterisks indicate significant difference between the wild type and *mgt6* mutant (Student’s *t*-test, ^∗^*P* < 0.05).

In a previous study, we have shown that knock-down of *MGT6* in transgenic plants by RNA interference resulted in growth retardation under low-Mg conditions (Mao et al., 2014). To further address the physiological role of MGT6, we isolated a previously unidentified T-DNA insertional mutant from the SALK collection (SALK_203866), in which the T-DNA insertion is located in the third exon of *MGT6*, 39 base pair (bp) upstream of the stop codon (**Figure 1A**). RT-PCR analyses showed that full-length *MGT6* transcript was not detectable in the *mgt6* mutant, while *MGT4* gene located in the same chromosome is normally expressed (**Figure 1B**). Consistent with earlier findings, mutation in *MGT6* leads to hypersensitivity to Mg limitation in that the *mgt6* mutants experienced growth defects at the germination stage (**Figures 1C–F**). When germinated on the medium containing no Mg^2+^ or 0.01 mM Mg^2+^, the *mgt6* mutants showed shorter roots and smaller and pale cotyledons (**Figures 1C,D**). In the presence of 0.25 mM Mg^2+^, *mgt6* seedlings appeared more normal, albeit still smaller than the wild-type (**Figure 1E**). Early seedling establishment during germination became comparable between wild-type and mutant plants when external Mg^2+^ reached 1.5 mM (**Figure 1F**). Statistical analysis of root length (**Figure 1G**) and seedling fresh weight (**Figure 1H**) verified the hypersensitivity to Mg deficiency in the *mgt6* mutant.

Because MGT-type transporters are capable of transporting several divalent cations in bacteria and yeast (Li et al., 2001; Mao et al., 2008), we examined the growth of *mgt6* mutant in the absence of other divalent cation nutrients. Whereas *mgt6* consistently displayed growth defects in the absence of Mg^2+^, seedling growth appeared indistinguishable between wild type and *mgt6* on the medium lacking other divalent cations including Ca^2+^, Fe^2+^, Mn^2+^, and Zn^2+^ (Supplementary Figure S1). These data suggest that under physiological conditions MGT6 may function in plants to cope with variable external Mg status, but is not relevant to other divalent cations.

### MGT6 Is Required for Plant Growth in *Arabidopsis* Under a Wide Range of External Mg^2+^ Concentrations

**FIGURE 2:**
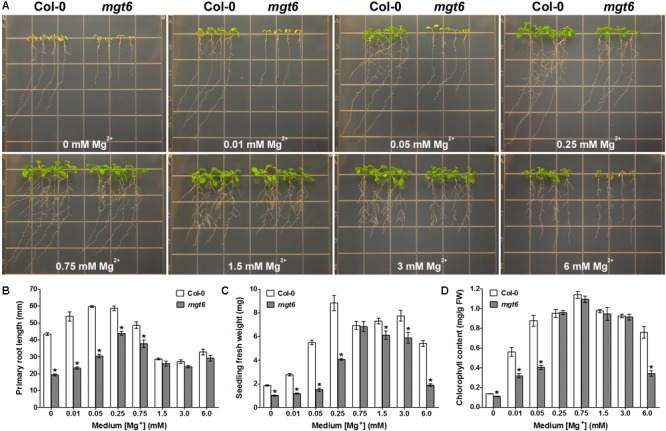
*mgt6* mutant is hypersensitive to Mg^2+^ deficiency and excess. **(A)** Growth phenotype of 5-day-old young seedlings transferred onto the medium supplemented with indicated concentrations of Mg^2+^ for 10 days. **(B)** Quantification of primary root length of the seedlings at the end of treatment. **(C)** Quantification of seedling fresh weight. **(D)** Quantification of leaf chlorophyll content. Data represent means ± SE of four replicate experiments. Asterisks indicate significant difference between the wild type and *mgt6* mutant (Student’s *t*-test, ^∗^*P* < 0.05).

To extend the phenotypic analysis of the *mgt6* mutant, we grew the seedlings of the mutant together with the wild-type plants on the plates containing various levels of Mg^2+^ in the post-germination assay. When grown on the low-Mg medium containing 0, 0.01, 0.05, or 0.25 mM Mg^2+^, the *mgt6* mutant plants were clearly stunted as compared with Col-0 (**Figure 2A**); the primary roots were shorter (**Figure 2B**) and the seedling fresh weight was significantly reduced (**Figure 2C**). Because Mg^2+^ is the central structural cation for chlorophyll, we analyzed the chlorophyll content in the young leaves and found that the mutant had a lower chlorophyll level under extremely low-Mg conditions (0, 0.01, and 0.05 mM Mg^2+^; **Figure 2D**). When the medium Mg^2+^ levels reached a moderate range (0.75, 1.25, and 3 mM), the growth of *mgt6* mutants appeared comparable to that of wild-type (**Figure 2A**), although primary root length or seedling fresh weight was slightly affected (**Figures 2B,C**). Notably, in the presence of 6 mM Mg^2+^ that is regarded as high, the *mgt6* seedlings exhibited a strong growth defect (**Figure 2A**), with much lower fresh weight (**Figure 2C**) and reduced chlorophyll content (**Figure 2D**) than wild-type plants. These data suggested that the *mgt6* mutant is not only compromised under low-Mg levels but also hypersensitive to high-Mg stress.

To verify the observed phenotypes in the *mgt6* mutant resulted from *MGT6* mutation, we conducted a complementation test. A genomic fragment of *MGT6* was introduced into the *mgt6* mutant. Several homozygous transgenic lines with a similar *MGT6* transcript level to that in wild type were obtained (Supplementary Figure S2B). Phenotypic analysis of two representative lines showed that seedling growth defects were fully rescued under both low- and high-Mg conditions (Supplementary Figure S2), suggesting MGT6 is indeed required for plant adaptation to Mg deficiency as well as plant tolerance to high-Mg stress.

**FIGURE 3:**
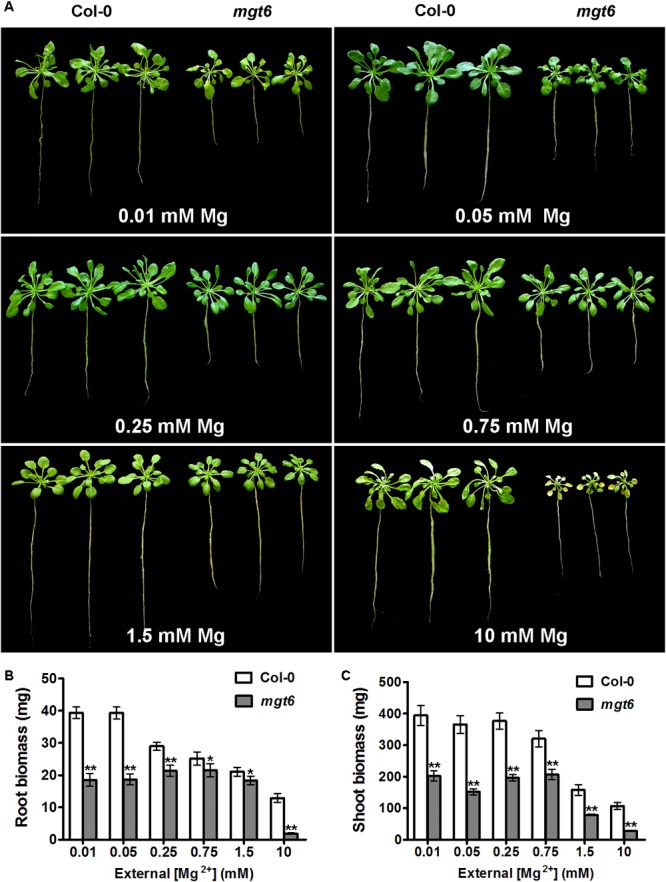
Mature plant phenotypes of *mgt6* mutants in a range of different external Mg^2+^ concentrations. **(A)** Growth phenotypes of 1-month-old wild-type plants and *mgt6* mutants under hydroponic conditions containing indicated concentrations of Mg^2+^. **(B)** Quantification of root biomass. **(C)** Quantification of shoot biomass. Data represent means ± SE of three replicate experiments. Asterisks indicate significant difference between the wild type and *mgt6* mutant (Student’s *t*-test, ^∗^*P* < 0.05, ^∗∗^*P* < 0.01).

To assess the function of MGT6 in mature plants, we grew wild-type and *mgt6* plants to flowering stage in the hydroponic solutions with defined levels of external Mg^2+^. We found that the *mgt6* plants showed compromised growth in all conditions tested (**Figure 3A**), but the growth difference was much more pronounced between wild-type and *mgt6* plants under extremely low (0.01 and 0.05 mM) and high-Mg^2+^ (10 mM) conditions, as revealed by the root and shoot biomass (**Figures 3B,C**). These results suggest that MGT6 is essential for plant growth at all developmental stages under a wide range of Mg^2+^ concentrations in the environment, and particularly plays an important role in plant adaption to low- and high-Mg^2+^ stresses.

### MGT6 Controls Plant Mg^2+^ Homeostasis in Both Root and Shoot Tissues

**FIGURE 4:**
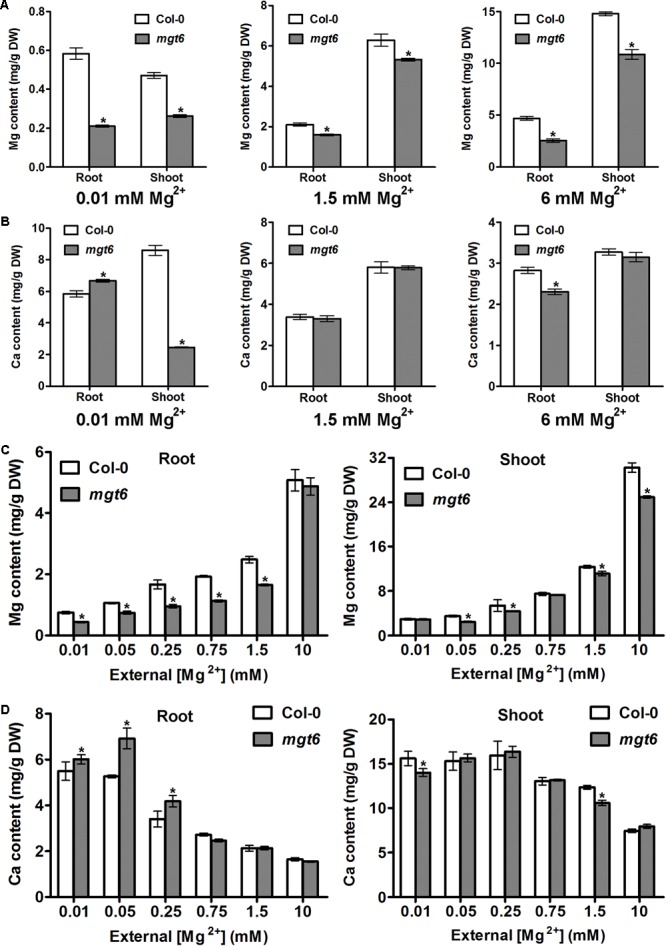
Mg and Ca content in the *mgt6* mutant under various growth conditions. **(A)** Mg content in the root and shoot of 2-week-old wild-type and *mgt6* plants grown on the plates containing indicated concentrations of Mg^2+^. **(B)** Ca content in the root and shoot of 2-week-old wild-type and *mgt6* plants grown on the plates containing indicated concentrations of Mg^2+^. **(C)** Mg content in the root and shoot of 4-week-old wild-type and *mgt6* plants grown in the hydroponic solutions containing various concentrations of Mg^2+^. **(D)** Ca content in the root and shoot of 4-week-old wild-type and *mgt6* plants grown in the hydroponic solutions containing various concentrations of Mg^2+^. Data represent means ± SE of four replicate experiments. Asterisks indicate significant difference between the wild type and *mgt6* mutant (Student’s *t*-test, ^∗^*P* < 0.05).

In order to investigate how plant Mg^2+^ homeostasis is affected by loss of *MGT6* function under various conditions, we measured metal content in the roots and shoots of the wild type and *mgt6*. We first employed the plant materials cultivated *in vitro* after 2 weeks’ growth on the plates. As expected, compared with wild-type plants, we observed a dramatic decrease in Mg content in both roots and shoots of *mgt6* mutants grown under low (0.01 mM) Mg conditions (**Figure 4A**). In the presence of normal (1.5 mM) and high (6 mM) external Mg^2+^ levels, *mgt6* mutants also contained less Mg in both roots and shoots than wild-type plants, when the seedlings were grown on the plates (**Figure 4A**). Because Ca is usually associated with Mg homeostasis, we also measured Ca content in the plants. While Ca content in the root of *mgt6* mutant was slightly higher, we surprisingly found that Mg deficiency resulted in a drastic reduction in shoot Ca compared with wild-type (**Figure 4B**). The Ca content, like other parameters of plant growth, was comparable between the wild-type and mutant plants grown under 1.5 mM Mg^2+^ (**Figure 4B**). The *mgt6* mutant retained significantly less Ca in the root and slightly decreased Ca content in the shoot tissue when plants were cultured in 6 mM Mg^2+^ (**Figure 4B**).

We further measured the Mg and Ca content in the hydroponically grown mature plants. As the external Mg^2+^ levels increased, wild-type plants accumulated elevated amount of Mg in both root and shoot tissues. The *mgt6* plants generally showed a significant reduction in root Mg content compared with wild type, except under 10 mM Mg^2+^ (**Figure 4C**). However, the shoot Mg content between wild type and *mgt6* is most strikingly different under 10 mM Mg^2+^, although under some other concentrations of Mg^2+^, such as 0.05, 0.25, and 1.5 mM, *mgt6* mutant also contained lower Mg content in the shoot compared with wild type (**Figure 4C**). Plant Ca contents are negatively correlated with external Mg^2+^ levels. Under low-Mg conditions (0.01, 0.05, and 0.25 mM), an obvious elevation in root Ca was observed in the *mgt6* mutant (**Figure 4D**), presumably due to the antagonistic interaction between Mg and Ca. These results suggest MGT6 regulates plant Mg^2+^ homeostasis in both roots and shoots, and functions in a wide range of external Mg^2+^ concentrations at all developmental stages.

### Grafting Assay Uncovers a Shoot-Based Mechanism for MGT6 Function in High-Mg Tolerance

**FIGURE 5:**
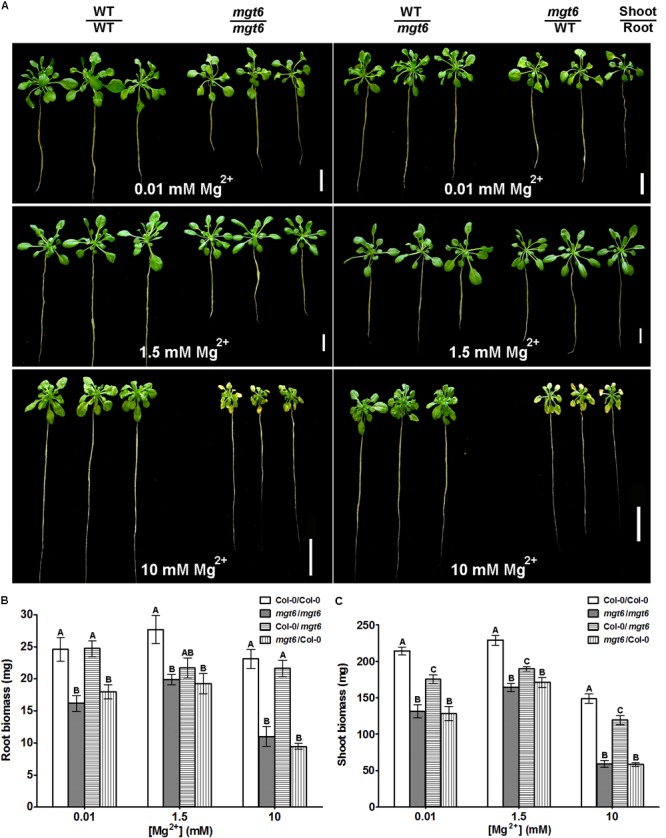
Phenotypic analysis of reciprocal grafting of wild-type and *mgt6* plants. **(A)** Growth phenotypes of 1-month-old grafted plants with different combinations under hydroponic conditions containing indicated concentrations of Mg^2+^. The genotype of the shoot scion is indicated in the upper part and the genotype of the rootstock is indicated in the lower part. Scale bar = 2 cm. **(B)** Quantification of root biomass. **(C)** Quantification of shoot biomass. Data represent means ± SE of three replicate experiments. Columns with different letters indicate significant difference (one-way ANOVA test, *P* < 0.05).

**FIGURE 6:**
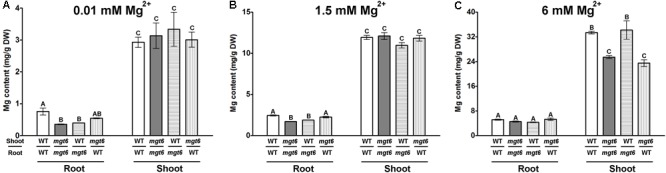
Mg content in grafted plants under different external Mg^2+^ conditions. **(A–C)** Determination of Mg content in the root and shoot tissues of four types of grafted plants grown in the hydroponic solutions containing different concentrations of Mg^2+^ (0.01, 1.5, and 6 mM). Data represent means ± SE of four replicate experiments. Columns with different letters indicate significant difference (one-way ANOVA test, *P* < 0.05).

While the low-Mg sensitive phenotype of *mgt6* can be explained by impaired Mg^2+^ uptake by root under Mg-limited conditions in the mutant, the high-Mg susceptibility of *mgt6* remains obscure. Since MGT6 controls both root and shoot Mg^2+^ homeostasis, we attempted to further investigate the mechanism by which MGT6 contributes to plant Mg^2+^ tolerance. Because MGT6 is widely expressed in plants, we decided to examine the relative contribution of MGT6 in roots versus in shoots through reciprocal grafting experiments between *mgt6* mutants and wild-type plants (**Figure 5**). When grown under low-Mg^2+^ conditions (0.01 mM), the shoots with wild-type scions and *mgt6* rootstocks appeared to be smaller than that of self-grafted wild-type plants, although the root looked similar. The grafted plants with *mgt6* scions and wild-type rootstocks were significantly smaller than wild-type self-grafted plants, but generally larger than *mgt6* self-grafted plants. Under the moderate level of Mg^2+^ (1.5 mM), both groups of the reciprocal grafted plants grew smaller than wild-type self-grafted plants. However, in the hydroponic culture containing 10 mM Mg^2+^, which is considered to be a toxic concentration, the grafted plants with *mgt6* scions and wild-type rootstocks phenocopied the defects seen in the *mgt6* self-grafts, whereas the grafted plants with wild-type scions and *mgt6* rootstocks resembled the phenotype of wild-type self-grafted plants (**Figure 5A**). We measured root and shoot fresh weight quantitatively, which verified the growth phenotypes (**Figures 5B,C**). These observations suggested that MGT6 is important in both root and shoot tissues when external Mg^2+^ is low and moderate. Presumably, MGT6-mediated absorption of external Mg^2+^ represents the dominant role under these conditions. When the external Mg^2+^ is extremely high, MGT6 function in the shoot becomes critical to detoxify excessive Mg^2+^ at the whole plant level. Consistent with this notion, wild-type scions grafted on mgt6 rootstocks lead to significantly lower root Mg^2+^ content under 0.01 and 1.5 mM Mg^2+^ conditions (**Figures 6A,B**). In the presence of 10 mM external Mg^2+^, shoots from *mgt6* grafted onto wild-type rootstocks retained much less Mg^2+^ in the shoot, similar to that observed in self-grafted *mgt6* plants (**Figure 6C**). This further supported the idea that MGT6 fulfills a shoot-based mechanism to detoxify excessive Mg^2+^, which could involve vacuolar Mg^2+^ storage based on the observation of lower Mg content in the mutant shoots.

### Functional Synergy of MGT6 and MGT7 in *Arabidopsis*

*Arabidopsis MGT7* encodes a low-affinity Mg^2+^ transporter (Mao et al., 2008) and is indispensable for optimal plant growth under low-Mg^2+^ conditions (Gebert et al., 2009). To investigate the functional interaction between MGT6 and MGT7, we created a double mutant that lacks both *MGT6* and *MGT7* transcripts (Supplementary Figure S3A). We found that the *mgt6 mgt7* double mutant displayed pronounced growth retardation in the soil (Supplementary Figure S3B). Quantitative analysis indicated that the shoot fresh weight of the double mutant was only half of that of the wild type and single mutants (Supplementary Figure S3C).

**FIGURE 7:**
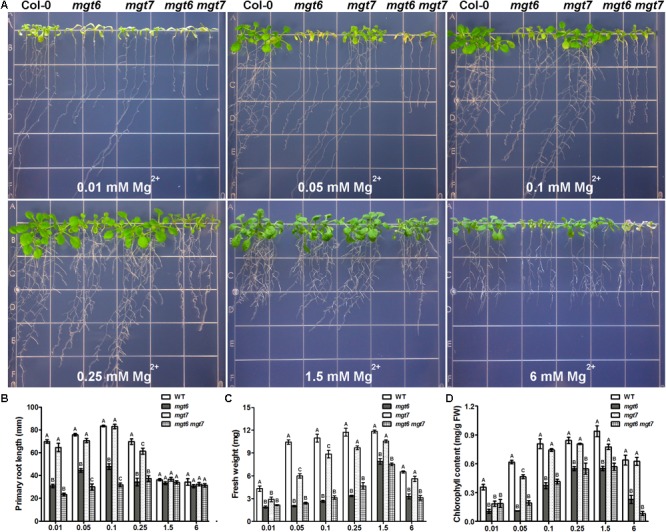
The *mgt6 mgt7* double mutant shows enhanced sensitivity to low- and high-Mg stresses than the single mutants. **(A)** Growth phenotype of 5-day-old young seedlings transferred onto the medium supplemented with indicated concentrations of Mg^2+^ for 10 days. **(B)** Quantification of primary root length of 15-day-old seedlings at the end of treatment. **(C)** Quantification of seedling fresh weight. **(D)** Quantification of leaf chlorophyll content. Data represent means ± SE of four replicate experiments. Columns with different letters indicate significant difference (one-way ANOVA test, *P* < 0.05).

We examined the growth phenotype of *mgt6 mgt7* double mutant under various external Mg^2+^ concentrations, in comparison with wild-type as well as the *mgt6* and *mgt7* single mutants. While *mgt6* single mutants exhibited very strong growth defects under both low- and high-Mg conditions, the phenotype of *mgt7* single mutant under the same condition was mild (**Figure 7A**). However, the *mgt6 mgt7* double mutant was significantly more sensitive to external Mg^2+^ than the *mgt6* single mutant (**Figure 7A**). The primary root of *mgt6 mgt7* was shorter than that of *mgt6* under low-Mg conditions (**Figure 7B**), although seedling fresh weight was comparable (**Figure 7C**). The leaf chlorophyll content in *mgt6 mgt7*was lower compared with *mgt6* when high Mg^2+^ is present in the medium (**Figure 7D**).

Gene expression analysis indicated that a handful of gene markers (Kamiya et al., 2012) were more responsive to Mg-starvation in the *mgt6* or *mgt7* mutant background than in the wild type, suggesting that the *mgt6* and *mgt7* mutants are impaired in low-Mg^2+^ adaptation. Consistent with the more severe phenotype, the *mgt6 mgt7* double mutant displayed enhanced expression of Mg-starvation marker genes compared with the single mutants (Supplementary Figure S4). Taken together, these results indicate both MGT6 and MGT7 are important for plant Mg homeostasis and their functions are additive in regulating Mg^2+^ transport under a wide range of external Mg^2+^ concentrations.

## Discussions

In addition to air and water, plant growth and development rely on mineral nutrients taken up by roots and translocated into the shoot tissues through apoplast and symplast pathways, which entail not only transpiration-driven mass flow but also active membrane transport processes facilitated by various ion channels and transporters. Mg is an essential macronutrient in plants with diverse biological functions. However, the molecular mechanisms for Mg transport and homeostasis in plant cells remain largely unknown. Genomes of many plants such as *Arabidopsis*, rice, and maize, encode homologs of the bacterial CorA-type proteins referred to as MGTs/MRS2s (Li et al., 2001, 2016; Saito et al., 2013). Some members of the MGT family have been functionally characterized, but the physiological roles of these transporters are not well understood. In *Arabidopsis*, we previously showed that MGT6 is capable of facilitating high-affinity Mg^2+^ uptake from the soil when external Mg^2+^ concentration is in the sub-millimolar range (Mao et al., 2014). Consistent with this role, expression of the *MGT6* gene is highly inducible in the root tissues in response to low Mg (Mao et al., 2014). In the present study, we not only corroborated earlier findings regarding the critical role of MGT6 in low-Mg adaptation, but also extended the function of MGT6 in controlling plant Mg^2+^ homeostasis within a wide range of external Mg^2+^ levels. The *mgt6* knock-out mutant displayed obvious phenotype under high-Mg conditions, suggesting MGT6 exerts physiological functions in plants other than Mg^2+^ absorption. In higher plants, after absorption from the soil solution by roots, Mg^2+^ is believed to be transported to the aerial parts via transpiration stream moving through the xylem vessels. However, little is known about the molecular identity of the transporters involved in this long-distance transport. MGT6 might fulfill such a role in the xylem transport of Mg^2+^. Considering the negative membrane potential, Mg^2+^ is expected to be loaded passively into the pericycle cells. MGT6 may be responsible for Mg^2+^ import into pericycle and xylem parenchyma cells. On the other hand, the possibility that MGT6 serves as an “exporter” in this process cannot be excluded. It is generally believed that ion secretion occurs across plasmalemma of the parenchyma cells surrounding the xylem vessels (Clarkson, 1993; Gaymard et al., 1998). Interestingly, MGT5, the closest homolog of MGT6 in *Arabidopsis*, was shown to be a bidirectional Mg^2+^ carrier that operates in a concentration-dependent manner (Li et al., 2008). Therefore, it is possible that MGT6 might also function in Mg^2+^ efflux from xylem parenchyma cells, pushing Mg^2+^ influx into the xylem vessel. Future electrophysiological analysis of MGT6 conductance is required to test this hypothesis.

Although Mg^2+^ is an essential mineral, high levels of Mg^2+^, such as those in the serpentine soils, could be toxic to plants. Recently we established that vacuolar sequestering of Mg^2+^, regulated by the tonoplast CBL-CIPK signaling network, is crucial for plants to survive under high-Mg conditions (Tang et al., 2015). In the present work, we uncovered another component mainly fulfilled by MGT6 that underlies high-Mg tolerance at the whole plant level. Previous studies indicate that serpentine-adapted plants appear to efficiently transport Mg^2+^ from root to shoot, whereas the serpentine-sensitive counterparts are less capable of driving Mg^2+^ entry into the transpiration stream, resulting in a lower Mg^2+^ concentration in the shoot (Palm et al., 2012). Consistent with this finding, our physiological analysis of *mgt6* mutant under high-Mg conditions showed that *mgt6* retained considerably less Mg^2+^ in the shoot tissue compared to wild-type, accompanied by the growth retardation upon high-Mg stress. These data support the notion that long-distance Mg^2+^ transport mediated by MGT6 may play a critical role in protecting plants from Mg^2+^ toxicity at the whole plant level. More importantly, reciprocal grafting test indicated that MGT6 function in the shoot tissue is responsible for the high-Mg^2+^ tolerance. Considering the plasma membrane localization of MGT6, it is reasonable to speculate that MGT6 probably facilitates Mg^2+^ entry into the cytosol of leaf cells after the Mg^2+^ ions in the xylem unload into the apoplastic space. The excessive Mg^2+^ in the cytosol is subsequently sequestered into the central vacuole via tonoplast-localized Mg^2+^ transporters. This transport cascade is critical for detoxification of excessive Mg^2+^, which is reminiscent of a recent model proposed for Ca^2+^ detoxification in plants (Wang et al., 2017).

Another notable finding in this study is that another MGT-type transporter MGT7 partially overlaps with the function of MGT6. With a preferential expression in the root, MGT7 was shown to be important for plant adaptation to low-Mg conditions (Gebert et al., 2009). In our study, we found that although MGT6 plays a more dominant role in low-Mg conditions, MGT7 seems to be additive to MGT6 function because *mgt6 mgt7* double mutant is more sensitive to low-Mg stress than the *mgt6* single mutant, which is also supported by the enhanced activation of Mg-starvation gene expression in the double mutant. Interestingly, under high-Mg conditions, mutation of MGT7 also significantly enhanced the sensitivity of *mgt6*, although single mutant of *mgt7* only exhibited a subtle phenotype under the same condition. These results suggest that MGT7 synergistically works together with MGT6 in the context of Mg^2+^ homeostasis at the whole plant level. Further investigations will sort out the mode of action for each of them to explain this functional synergy.

Subcellular localization of the MGT proteins may prove to be difficult to study. For instance, recent studies reported discrepant cellular localizations for MGT6 in the plasma membrane (Mao et al., 2014) and endoplasmic reticulum (ER; Oda et al., 2016), respectively. Several other MGT members such as MGT7 (Gebert et al., 2009) and MGT4 (Li et al., 2015) were also shown to be ER-associated, which needs to be re-evaluated because quite a few membrane proteins tend to be mis-targeted to ER, especially when overexpressed in a transient expression system (Denecke et al., 2012; Quattrocchio et al., 2013; Segami et al., 2014). Future studies using the native promoter, coupled with functional complementation in the mutant background as well as other approaches, are needed to verify the subcellular localization of MGT-type transporters *in situ*. It will also be interesting to examine if the targeting of MGT6 or MGT7 would be dynamically altered in different subcellular compartments in response to various Mg^2+^ concentrations.

As sessile organisms, plants have to cope with fluctuating concentrations of Mg^2+^ in nature. How plants maintain a balanced level of Mg^2+^ is not well understood. The present study as well as our previous work provides a working model in which MGT6 plays a dual role in controlling Mg^2+^ homeostasis. When external Mg^2+^ is limited, expression of *MGT6* is induced in root epidermal cells and root hairs, making this transporter primarily responsible for Mg^2+^ uptake from the soil. When external Mg^2+^ is sufficient or becomes excessive, MGT6 mediates Mg^2+^ loading into the shoot tissues, where leaf mesophyll cells can subsequently sequester extra amount of Mg^2+^ into large vacuoles via yet-unknown transporters. Future efforts should be made in identifying uncharacterized Mg^2+^ transport proteins in plants. Furthermore, establishing the regulators and signaling pathways that fine-tune the expression and function of these transport systems will be a challenging but urgent task, which will ultimately lead to genetic manipulation of plants for precise adaption to the changing Mg^2+^ concentrations in the environment.

## Author Contributions

Y-WY and R-JT designed and conducted most of the experiments, interpreted the results, and wrote the draft of the manuscript. D-DM, X-XZ, Q-LT, and Y-PL assisted in some experiments and helped analyze the data. LY and J-LQ provided tools and reagents and made helpful discussions. SL supervised and conceptualized the study and finalized the paper. All the authors approved the final version of the manuscript.

## Conflict of Interest Statement

The authors declare that the research was conducted in the absence of any commercial or financial relationships that could be construed as a potential conflict of interest.

## References

[B1] Brady K. U. Kruckeberg A. R. Bradshaw H. D. (2005). Evolutionary ecology of plant adaptation to serpentine soils. *Annu. Rev. Ecol. Evol. Syst.* 36 243–266. 10.1146/annurev.ecolsys.35.021103.105730

[B2] Chen J. Li L. G. Liu Z. H. Yuan Y. J. Guo L. L. Mao D. D. (2009). Magnesium transporter AtMGT9 is essential for pollen development in *Arabidopsis*. *Cell Res.* 19 887–898. 10.1038/cr.2009.58 19436262

[B3] Chen Z. C. Yamaji N. Horie T. Che J. Li J. An G. (2017). A Magnesium transporter *OsMGT1* plays a critical role in salt tolerance in rice. *Plant Physiol.* 174 1837–1849. 10.1104/pp.17.00532 28487477 PMC5490922

[B4] Chen Z. C. Yamaji N. Motoyama R. Nagamura Y. Ma J. F. (2012). Up-regulation of a magnesium transporter gene OsMGT1 is required for conferring aluminum tolerance in rice. *Plant Physiol.* 159 1624–1633. 10.1104/pp.112.199778 22732245 PMC3425201

[B5] Clarkson D. T. (1993). Roots and the delivery of solutes to the xylem. *Philos. Trans. R. Soc. B* 341 5–17. 10.1098/rstb.1993.0086

[B6] Clough S. J. Bent A. F. (1998). Floral dip: a simplified method for *Agrobacterium*-mediated transformation of *Arabidopsis thaliana*. *Plant J.* 16 735–743. 10.1046/j.1365-313x.1998.00343.x 10069079

[B7] Conn S. J. Conn V. Tyerman S. D. Kaiser B. N. Leigh R. A. Gilliham M. (2011). Magnesium transporters, MGT2/MRS2-1 and MGT3/MRS2-5, are important for magnesium partitioning within *Arabidopsis thaliana* mesophyll vacuoles. *New Phytol.* 190 583–594. 10.1111/j.1469-8137.2010.03619.x 21261624

[B8] Denecke J. Aniento F. Frigerio L. Hawes C. Hwang I. Mathur J. (2012). Secretory pathway research: the more experimental systems the better. *Plant Cell* 24 1316–1326. 10.1105/tpc.112.096362 22523202 PMC3398477

[B9] Drummond R. S. M. Tutone A. Li Y. C. Gardner R. C. (2006). A putative magnesium transporter AtMRS2-11 is localized to the plant chloroplast envelope membrane system. *Plant Sci.* 170 78–89. 10.1016/j.plantsci.2005.08.018

[B10] Eshaghi S. Niegowski D. Kohl A. Molina D. M. Lesley S. A. Nordlund P. (2006). Crystal structure of a divalent metal ion transporter CorA at 2.9 angstrom resolution. *Science* 313 354–357. 10.1126/science.1127121 16857941

[B11] Gaymard F. Pilot G. Lacombe B. Bouchez D. Bruneau D. Boucherez J. (1998). Identification and disruption of a plant shaker-like outward channel involved in K+ release into the xylem sap. *Cell* 94 647–655. 10.1016/S0092-8674(00)81606-2 9741629

[B12] Gebert M. Meschenmoser K. Svidova S. Weghuber J. Schweyen R. Eifler K. (2009). A root-expressed magnesium transporter of the MRS2/MGT gene family in *Arabidopsis thaliana* allows for growth in low-Mg2+ environments. *Plant Cell* 21 4018–4030. 10.1105/tpc.109.070557 19966073 PMC2814501

[B13] Hermans C. Conn S. J. Chen J. G. Xiao Q. Y. Verbruggen N. (2013). An update on magnesium homeostasis mechanisms in plants. *Metallomics* 5 1170–1183. 10.1039/c3mt20223b 23420558

[B14] Kamiya T. Yamagami M. Hirai M. Y. Fujiwara T. (2012). Establishment of an *in planta* magnesium monitoring system using *CAX3* promoter-luciferase in *Arabidopsis*. *J. Exp. Bot.* 63 355–363. 10.1093/jxb/err283 21914662 PMC3245472

[B15] Li H. Y. Du H. M. Huang K. F. Chen X. Liu T. Y. Gao S. B. (2016). Identification, and functional and expression analyses of the CorA/MRS2/MGT-Type magnesium transporter family in maize. *Plant Cell Physiol.* 57 1153–1168. 10.1093/pcp/pcw064 27084594

[B16] Li J. Huang Y. Tan H. Yang X. Tian L. Luan S. (2015). An endoplasmic reticulum magnesium transporter is essential for pollen development in *Arabidopsis*. *Plant Sci.* 231 212–220. 10.1016/j.plantsci.2014.12.008 25576006

[B17] Li L. Tutone A. F. Drummond R. S. Gardner R. C. Luan S. (2001). A novel family of magnesium transport genes in Arabidopsis. *Plant Cell* 13 2761–2775. 10.1105/tpc.13.12.2761 11752386 PMC139487

[B18] Li L. G. Sokolov L. N. Yang Y. H. Li D. P. Ting J. Pandy G. K. (2008). A mitochondrial magnesium transporter functions in *Arabidopsis* pollen development. *Mol. Plant* 1 675–685. 10.1093/mp/ssn031 19825572

[B19] Liang S. Qi Y. F. Zhao J. Li Y. F. Wang R. Shao J. X. (2017). Mutations in the Arabidopsis *AtMRS2-11/AtMGT10/VAR5* gene cause leaf reticulation. *Front. Plant Sci.* 8:2007. 10.3389/fpls.2017.02007 29234332 PMC5712471

[B20] Lunin V. V. Dobrovetsky E. Khutoreskaya G. Zhang R. Joachimiak A. Doyle D. A. (2006). Crystal structure of the CorA Mg2+ transporter. *Nature* 440 833–837. 10.1038/nature04642 16598263 PMC3836678

[B21] Mao D. Chen J. Tian L. Liu Z. Yang L. Tang R. (2014). *Arabidopsis* transporter MGT6 mediates magnesium uptake and is required for growth under magnesium limitation. *Plant Cell* 26 2234–2248. 10.1105/tpc.114.124628 24794135 PMC4079380

[B22] Mao D. D. Tian L. F. Li L. G. Chen J. Deng P. Y. Li D. P. (2008). *AtMGT7*: an *Arabidopsis* gene encoding a low-affinity magnesium transporter. *J. Integr. Plant Biol.* 50 1530–1538. 10.1111/j.1744-7909.2008.00770.x 19093971

[B23] Marsch-Martínez N. Franken J. Gonzalez-Aguilera K. L. De Folter S. Angenent G. Alvarez-Buylla E. R. (2013). An efficient flat-surface collar-free grafting method for *Arabidopsis thaliana* seedlings. *Plant Methods* 9:14. 10.1186/1746-4811-9-14 23641687 PMC3668283

[B24] Murashige T. Skoog F. (1962). A revised medium for rapid growth and bioassays with tobacco tissue cultures. *Physiol. Plant* 15 473–495. 10.1111/j.1399-3054.1962.tb08052.x

[B25] Oda K. Kamiya T. Shikanai Y. Shigenobu S. Yamaguchi K. Fujiwara T. (2016). The Arabidopsis Mg transporter, MRS2-4, is essential for Mg homeostasis under both low and high Mg conditions. *Plant Cell Physiol.* 57 754–763. 10.1093/pcp/pcv196 26748081

[B26] Palm E. Brady K. Van Volkenburgh E. V. (2012). Serpentine tolerance in *Mimulus guttatus* does not rely on exclusion of magnesium. *Funct. Plant Biol.* 39 679–688. 10.1007/s00442-009-1448-0 32480819

[B27] Quattrocchio F. M. Spelt C. Koes R. (2013). Transgenes and protein localization: myths and legends. *Trends Plant Sci.* 18 473–476. 10.1016/j.tplants.2013.07.003 23932488

[B28] Saito T. Kobayashi N. I. Tanoi K. Iwata N. Suzuki H. Iwata R. (2013). Expression and functional analysis of the CorA-MRS2-ALR-type magnesium transporter family in rice. *Plant Cell Physiol.* 54 1673–1683. 10.1093/pcp/pct112 23926064

[B29] Schock I. Gregan J. Steinhauser S. Schweyen R. Brennicke A. Knoop V. (2000). A member of a novel *Arabidopsis thaliana* gene family of candidate Mg2+ ion transporters complements a yeast mitochondrial group II intron-splicing mutant. *Plant J.* 24 489–501. 10.1046/j.1365-313x.2000.00895.x 11115130

[B30] Segami S. Makino S. Miyake A. Asaoka M. Maeshima M. (2014). Dynamics of vacuoles and H^+^-pyrophosphatase visualized by monomeric green fluorescent protein in *Arabidopsis*: artifactual bulbs and native intravacuolar spherical structures. *Plant Cell* 26 3416–3434. 10.1105/tpc.114.127571 25118245 PMC4371836

[B31] Shaul O. (2002). Magnesium transport and function in plants: the tip of the iceberg. *Biometals* 15 309–323. 10.1023/A:1016091118585 12206396

[B32] Sun Y. Yang R. A. Li L. G. Huang J. R. (2017). The magnesium transporter MGT10 is essential for chloroplast development and photosynthesis in *Arabidopsis thaliana*. *Mol. Plant* 10 1584–1587. 10.1016/j.molp.2017.09.017 28989088

[B33] Szegedy M. A. Maguire M. E. (1999). The CorA Mg^2+^ transport protein of *Salmonella typhimurium* mutagenesis of conserved residues in the second membrane domain. *J. Biol. Chem.* 274 36973–36979. 10.1074/jbc.274.52.36973 10601252

[B34] Tang R. J. Luan S. (2017). Regulation of calcium and magnesium homeostasis in plants: from transporters to signaling network. *Curr. Opin. Plant Biol.* 39 97–105. 10.1016/j.pbi.2017.06.009 28709026

[B35] Tang R. J. Zhao F. G. Garcia V. J. Kleist T. J. Yang L. Zhang H. X. (2015). Tonoplast CBL-CIPK calcium signaling network regulates magnesium homeostasis in Arabidopsis. *Proc. Natl. Acad. Sci. U.S.A.* 112 3134–3139. 10.1073/pnas.1420944112 25646412 PMC4364200

[B36] Turner T. L. Bourne E. C. Von Wettberg E. J. Hu T. T. Nuzhdin S. V. (2010). Population resequencing reveals local adaptation of *Arabidopsis lyrata* to serpentine soils. *Nat. Genet.* 42 260–263. 10.1038/ng.515 20101244

[B37] Wang Y. Kang Y. Ma C. Miao R. Wu C. Long Y. (2017). CNGC2 is a Ca2+ influx channel that prevents accumulation of apoplastic Ca2+ in the leaf. *Plant Physiol.* 173 1342–1354. 10.1104/pp.16.01222 27999084 PMC5291024

[B38] Xu X. F. Wang B. Lou Y. Han W. J. Lu J. Y. Li D. D. (2015). *Magnesium transporter 5* plays an important role in Mg transport for male gametophyte development in *Arabidopsis*. *Plant J.* 84 925–936. 10.1111/tpj.13054 26478267

