# Supplementary material for: Magnesium Transporter MGT6 Plays an Essential Role in Maintaining Magnesium Homeostasis and Regulating High Magnesium Tolerance in Arabidopsis
Source: Front Plant Sci. 2018 Mar 12;9:274. doi: 10.3389/fpls.2018.00274 (PMC5857585; doi:10.3389/fpls.2018.00274)
Supplement: Supplementary file 1 [file Data_Sheet_1.PDF]

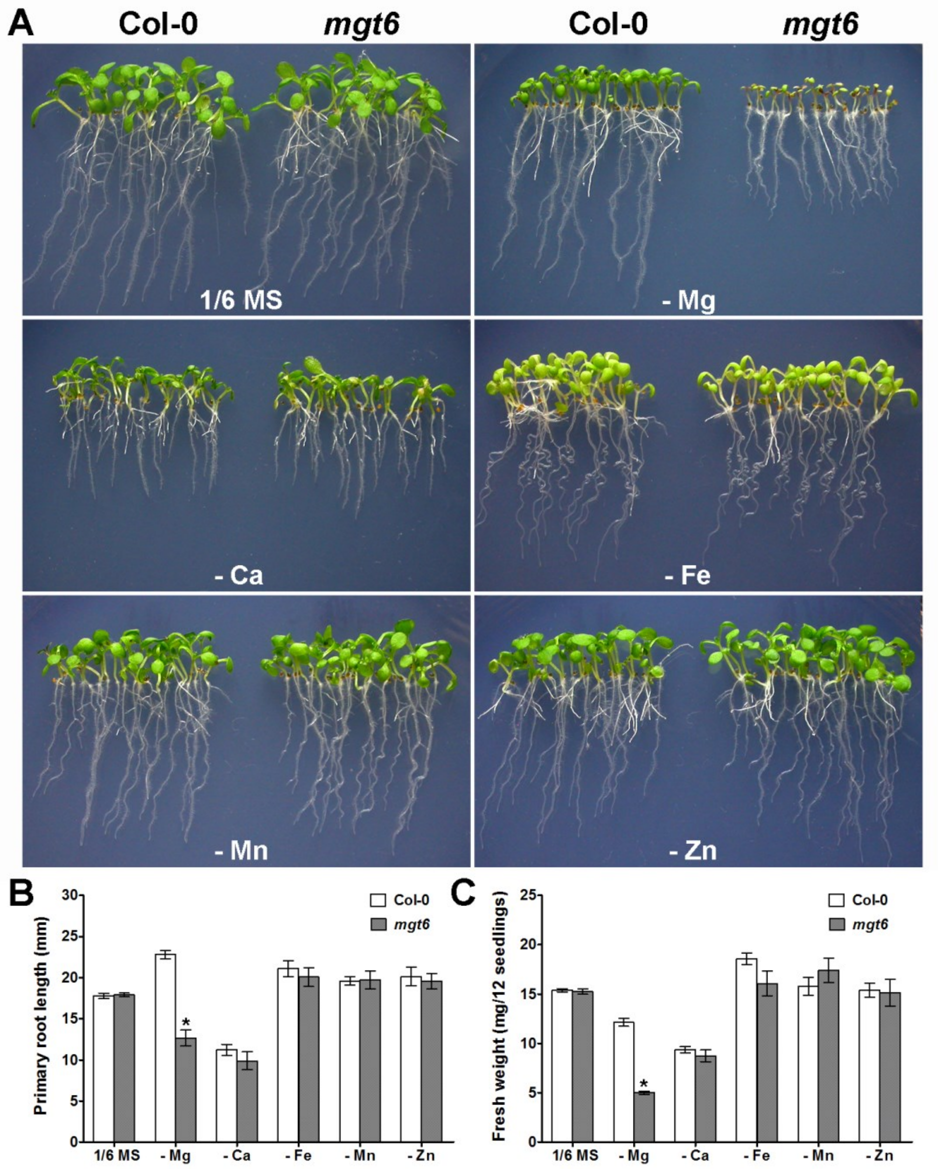

FIGURE S1 | The *mgt6* mutant is specifically hypersensitive to Mg^{2+} deficiency but not to the lack of other divalent cations. (A) Growth phenotype of young seedlings 7 days after seed germination on 1/6 MS medium or medium lacking a particular divalent cation as indicated. **(B)** Quantification of primary root length of 6-day-old seedlings. **(C)** Quantification of seedling fresh weight. Data represent means ± SE of four replicate experiments. Asterisks indicate significant difference from the wild type (Student's t test, $*P < 0.05$).

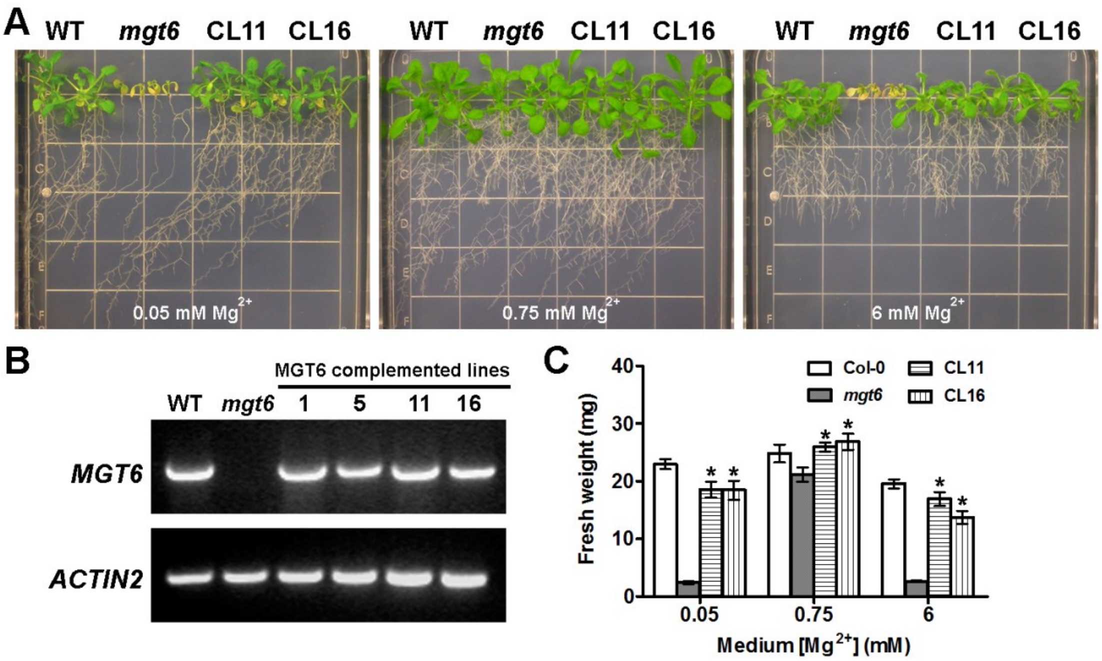

FIGURE S2 | Expression of *MGT6* locus in the *mgt6* mutant restores the growth defects under both low and high Mg conditions. (A) Growth phenotype of 5-day-old young transferred onto the medium supplemented with indicated concentrations of Mg^{2+} for 12 days. The *MGT6*-complemented lines CL11 and CL16 considerably rescued the phenotype of *mgt6* mutant. **(B)** RT-PCR analysis of *MGT6* in wild-type, *mgt6* mutant and several *mgt6* transgenic lines expressing *MGT6*. **(C)** Quantification of seedling fresh weight. Data represent means ± SE of four replicate experiments. Asterisks indicate significant difference from the *mgt6* mutant (Student's t test, $^*P < 0.05$).

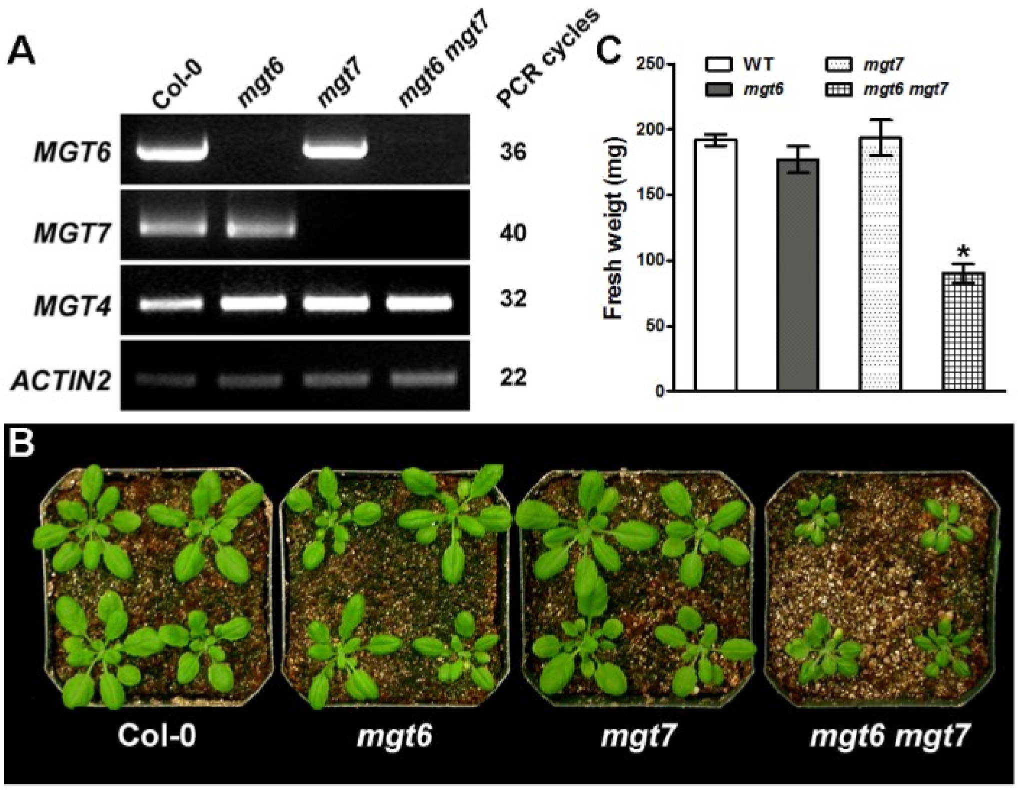

FIGURE S3 | Generation and growth phenotype of the *mgt6 mgt7* double mutant. (A) RT-PCR analysis of *MGT6*, *MGT7* and *MGT4* gene expression in wild-type, *mgt6*, *mgt7* and *mgt6 mgt7* seedlings. **(B)** 4-week-old wild-type and mutant plants grown in the soil. The *mgt6 mgt7* double mutants displayed pronounced growth retardation. **(C)** Quantification of plant shoot biomass. Data represent means ± SE of four replicate experiments. Asterisk indicates significant difference from the wild type as well as the *mgt6* and *mgt7* single mutants (Student's t test, **P* < 0.05).

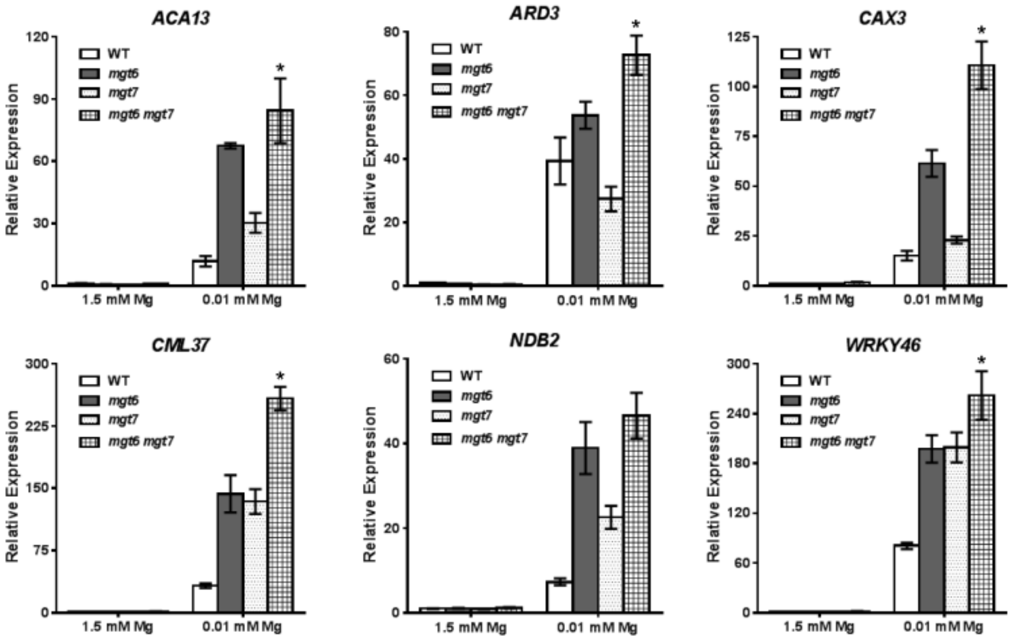

FIGURE S4 | Expression of Mg-starvation marker genes in single and double *mgt6 mgt7* mutants. Quantitative real-time PCR analysis of *ACA13* (AT3G22910), *ARD3* (AT2G26400), *CAX3* (AT3G51860), *CML37* (AT5G42380), *NDB2* (AT4G05020) and *WRKY46* (AT2G46400) in response to low-Mg starvation in the wild-type, *mgt6*, *mgt7* and *mgt6 mgt7* seedlings. The relative expression of each gene was double normalized using the housekeeping gene *ACTIN2* (AT3G18780) and using the control expression values measured in the wild type at 1.5 mM Mg. Data represents mean ± SD (n=4). Asterisks indicate significant difference from the *mgt6* mutant (Student's t test, *P < 0.05).
